# Assessing the acceptability of technological health innovations in sub-Saharan Africa: a scoping review and a best fit framework synthesis

**DOI:** 10.1186/s12913-023-09897-4

**Published:** 2023-08-31

**Authors:** Sarah Louart, Gildas Boris Hedible, Valéry Ridde

**Affiliations:** 1 Univ. Lille, CNRS, UMR 8019 - CLERSE - Centre Lillois d’Etudes Et de Recherches Sociologiques Et Economiques, 59000 Lille, France; 2 ALIMA, the Alliance for International Medical Action, Dakar, Senegal; 3 CERPOP- UMR 1295 - Inserm, Université Paul Sabatier 3, Toulouse, France; 4 Université Paris Cité, IRD, INSERM, Ceped, 75006 Paris, France; 5 Institut de Santé Et Développement, Université Cheikh Anta Diop, Dakar, Sénégal

**Keywords:** Acceptability, Innovation, Health, Scoping review, Best fit framework synthesis, Sub-Saharan Africa

## Abstract

Acceptability is a key concept used to analyze the introduction of a health innovation in a specific setting. However, there seems to be a lack of clarity in this notion, both conceptually and practically. In low and middle-income countries, programs to support the diffusion of new technological tools are multiplying. They face challenges and difficulties that need to be understood with an in-depth analysis of the acceptability of these innovations. We performed a scoping review to explore the theories, methods and conceptual frameworks that have been used to measure and understand the acceptability of technological health innovations in sub-Saharan Africa. The review confirmed the lack of common definitions, conceptualizations and practical tools addressing the acceptability of health innovations. To synthesize and combine evidence, both theoretically and empirically, we then used the "best fit framework synthesis" method. Based on five conceptual and theoretical frameworks from scientific literature and evidence from 33 empirical studies, we built a conceptual framework in order to understand the acceptability of technological health innovations. This framework comprises 6 determinants (compatibility, social influence, personal emotions, perceived disadvantages, perceived advantages and perceived complexity) and two moderating factors (intervention and context). This knowledge synthesis work has also enabled us to propose a chronology of the different stages of acceptability.

**Supplementary Information:**

The online version contains supplementary material available at 10.1186/s12913-023-09897-4.

## Background

Acceptability is a central concept reflecting a crucial process for understanding the effects of interventions in public health. As shown during the Covid-19 pandemic, the various responses to fighting the virus, such as the government measures taken, vaccination, technological tools used, etc., were accepted in varying degrees by the populations [1–5]. It is therefore important to measure and understand the acceptability of health interventions so as to adapt the actions implemented and achieve the health objectives targeted.

More specifically, concerning technological innovations, acceptability is often considered a crucial factor in the process of adoption, abandonment or diffusion of an innovation. Technological innovations historically have shaped the organization and transformation of societies, institutions and practices [6]. In the field of health, they are defined as “the application of organized knowledge and skills in the form of medicines, medical devices, vaccines, procedures and systems developed to solve a health problem and improve quality of life” [7]. To achieve these objectives for improving local and global health and social indicators, health technologies are subject to numerous technical evaluations before their dissemination: safety, ergonomics, effectiveness, checking of the results' validity, etc. However, users are not simply receivers of innovations, they have an active role in the innovation process, they are co-creators through their reaction, appropriation, diverted use, adaptation, etc. [8]. It is therefore also necessary to carry out a social assessment of these innovative health technologies in order to document the multiple factors that can influence their introduction and dissemination [9] and understand the processes that will ultimately lead to their adoption or abandonment. This issue is all the more important in low- and middle-income countries (LMIC) whose contexts often present additional challenges, such as unreliable power, poor internet and mobile signal connectivity, lack of transportation systems, medicine stockouts, lack of financial resources, conflict, health literacy, etc. [10, 11].

We are particularly interested in how to measure and understand the acceptability of an innovation in the context of the AIRE project (Améliorer l’Identification des détresses Respiratoires chez l’Enfant). It aims to introduce pulse oximeters (in the Integrated Management of Childhood Illness guidelines) in primary health centers in four West African countries (Burkina Faso, Guinea, Mali, Niger) to reduce mortality of children under five years old [12]. The pulse oximeter is considered here as a technological innovation in health. Thus, one of the AIRE research objectives is to measure the acceptability of this tool by health professionals and caregivers of children. We wanted to base this research on theories and analytical frameworks, as this is very important for ensuring the quality and rigor of the research [13]. We therefore initiated an informal literature review (with keywords related to theories, models, conceptual frameworks; as well as keywords related to acceptability and innovations), but it did not allow us to find a framework adapted to our study.

Our informal exploration of the literature concerning the concept of acceptability instead highlighted the diversity of terminologies or 'proxies' (acceptability, acceptance, adoption, satisfaction, willingness to use, feasibility, enjoyment, etc.) used to define the same concept, also noted by Bucyibaruta and colleagues [14]. While the criteria for assessing the quality of a concept are clarity (in a given context, the term should have only one meaning) and precision (the possibility of effectively distinguishing the empirical phenomena to which it applies from those to which it does not apply) [15], we found that the concept of acceptability does not meet either of these criteria. This conceptual grey area can create ambiguities regarding what is being measured or analyzed, prevent the development of appropriate data collection methods and tools [16] and make it difficult to make comparisons between studies and accumulate scientific knowledge [17]. Too often, the level of acceptability is measured in a simplistic way (agreement or disagreement with use or participation), without seeking to understand its determinants. However, there is a real distinction to be made between participation and acceptability, as shown in the article by Gooding et al. [18]. In their study, many factors influenced participation or non-participation, and it didn't necessarily match the opinion of the respondents.

Acceptability is a concept that is very complex to understand. It is not a simple binary decision, it is a dynamic process [19], determined by several factors and which varies over space and time [20]. Indeed, the level of acceptability of a health technology can evolve. Nadal et al. [17] also point out that most of the models used to measure acceptability do not take this temporality into account. They propose a "technology acceptance lifecycle" based on the evolution between "pre-use acceptability", "initial use acceptance" and "sustained use acceptance". Other authors, such as Sekhon et al. [21], incorporate a three-stage temporality into their model: prospective, concurrent and retrospective. Greenhalgh et al. [9] also capture this temporal evolution through the "continuous integration and adaptation over time" dimension of their framework.

There is a need to detail and circumscribe this concept to be able to produce a conceptual framework for measuring and understanding the acceptability of health innovations. To do so, it is first necessary to define the objective of this conceptualization, following the idea developed by Perski and Short [19]: “from a public health (as opposed to, for example, a philosophical) perspective, it can be argued that the utility of the concept of intervention acceptability lies in its ability to predict and explain key outcomes of interest”. We can therefore understand acceptability as how an actor reacts to a technological innovation, this reaction mechanism being itself influenced by multiple factors and contributing to determining the use (and/or agreement to use) of this technological innovation in health (without the two concepts of acceptability and use being blended).

The objective of our research is to propose a conceptual framework to help assess and understand the acceptability of technological innovations in health in sub-Saharan Africa.

## Methods

### Definition of innovation

We adopt the proposition formulated by Rogers [6], stating that “an innovation is an idea, practice, or object that is perceived as new by an individual or other unit of adoption”. However, we have focused on technological innovations (object, software, application, etc.), as we assumed that the acceptability of an idea, process, practice or technology was not necessarily determined by the same factors. Indeed, the literature argues that the acceptability of technological innovation can be influenced by the design [22], the ease of use of the tool [23], the level of technical expertise or any technical problems encountered [24]. We also chose to focus on innovations used in the context of a patient-caregiver interaction, rather than tools or software used only by patients (e.g. self-tests) or only by healthcare professionals (e.g. software designed to improve the organization of work in the health center). We hypothesized that the impact of the tool on the relationship and interactions between patient and provider may influence its acceptability [25]. Regarding the perception of the novelty of an object in relation to the context of its introduction, we adopted an emic point of view [26]. We considered as innovations the technologies that were described as such by the authors of the identified articles.

**Table:** 

To be considered an innovation in our study, there are three criteria:
An innovation is (i) a technological innovation (object, software, application, etc.), (ii) used in the context of a patient-caregiver interaction, and (iii) perceived and described as new in the context of its introduction

### Scoping review methodology

We first carried out a scoping review. We followed the steps proposed by Arksey and O'Malley [27]: (a) identifying the research question; (b) identifying relevant studies; (c) studies selection; (d) charting the data; and (e) summarizing and analyzing the results. We also followed the methodological improvements suggested by the VERDAS consortium (pilot round realization) [28]. The main question guiding our research was: "What theories, methods and conceptual frameworks are used to measure and understand the acceptability of technological innovations in health in sub-Saharan Africa?".

We used the search strategies presented in Appendix 1 to search the databases. Depending on each database, we used specific thesauri if available, and searched for our keywords in the abstracts and titles of articles. We ran our search equations in eight different databases during February 2020: PubMed, Scopus, EBSCOhost (Psychology and Behavioral Sciences Collection, Academic Search Premier, eBook Collection (EBSCOhost), EconLit, PsycArticles, PsychInfo, Business Source Complete), Web of Science, Cochrane (the whole Cochrane Library), Cairn, Opengrey, Scielo. All the collected references were registered in Zotero, which removed the duplicates. We then carried out a two-stage selection: first, we selected the articles based on their title and abstract and then based on their full text. The inclusion criteria were: the article (1) is in English, French or Spanish; (2) focuses on one or more sub-Saharan African country; (3) is about a technological innovation in health (as defined above); (4) its objective (secondary or main) is to study the acceptability of this innovation. The PRISMA flow diagram [29] was used to guide the selection process.

We then analyzed the bibliographies of the studies included in order to find any additional references. Articles that met all the criteria were then analyzed further. We used a checklist to extract the data of interest from each article. The extracted data were the characteristics of the article (authors, journal, year of publication, etc.); the characteristics of the innovation and the conditions of its introduction (country of introduction, type of innovation, target population, etc.); the definition, the theories or frameworks used (definition of acceptability, whether a theory or analytical framework was used, etc.); and finally, the evaluation (type of evaluation, data collection tools, main results, etc.).

### Best fit framework synthesis methodology

The scoping review could not identify a conceptual framework or theory (for an explanation of the difference between the two, see: [13]) relevant to our study, given the specificities of the context (sub-Saharan Africa), the field (health) and the type of innovation (see our definition). We therefore decided to use the best fit framework synthesis approach [30, 31] to construct a conceptual framework specific to the acceptability of health innovations. This approach assumes that existing frameworks do not necessarily fully match the subject of interest. However, they are the best that exist to date for producing an initial framework and study themes to be adapted, tested and improved with the empirical data of interest. Therefore, the approach proposes building on one or more existing framework to construct an 'a priori framework'. The best fit framework approach recommends combining several relevant models to provide a more complete basis for analysis, rather than arbitrarily choosing a single framework to serve as an a priori framework [31]. We have therefore based our work on the different frameworks identified through our scoping review [6, 23, 32, 33]. To this, we added two other frameworks not mentioned in the articles included in the scoping review but found during our informal exploration of the literature or through discussion within our team about the frameworks currently used in research to measure the acceptability of innovation [21, 34]. First, we built our a priori framework based on all the themes of the five frameworks identified. As recommended [31], we conducted a thematic analysis of the different dimensions of the five frameworks by identifying the commonalities and differences between the conceptual frameworks and theories and grouping them into themes (Appendix 2). We defined each of these themes to ensure a common understanding of the concepts and to facilitate subsequent coding of data. Then, following an abductive approach (back and forth between theoretical conjectures and empirical data), we confronted the different dimensions of the a priori framework with the empirical data from the articles identified through our scoping review.

The best fit framework method recommends carrying out a double literature review: the first identifies the conceptual frameworks or theories that can feed the formulation of the framework a priori, and the second collects the empirical data that will serve to improve the framework initially constituted [31]. In our case, our scoping review allowed us to identify both conceptual frameworks and empirical data, since our research was focused on the concept of acceptability, without being limited to either frameworks and theories or empirical data. We included protocols only to document the use of frameworks or theories to study acceptability, but then excluded them from further analysis since they do not present empirical data. We had also previously conducted an informal review of the literature to try to identify relevant frameworks or theories.

Empirical data on the subject of interest were therefore extracted from the results sections of the articles selected in our scoping review (excluding protocols). These data were then coded against the constructed themes of the a priori framework with Nvivo12. They were used as a basis for developing, improving, expanding, reducing or completing the themes of the framework initially built [30, 31]. Indeed, during this comparison between the a priori framework and the new data, the “Relationships between the themes of the framework are then either recreated or generated based on the evidence from the primary research studies included in the review” [31].

## Results

### What the scoping review tells us about acceptability

**Fig. 1:**
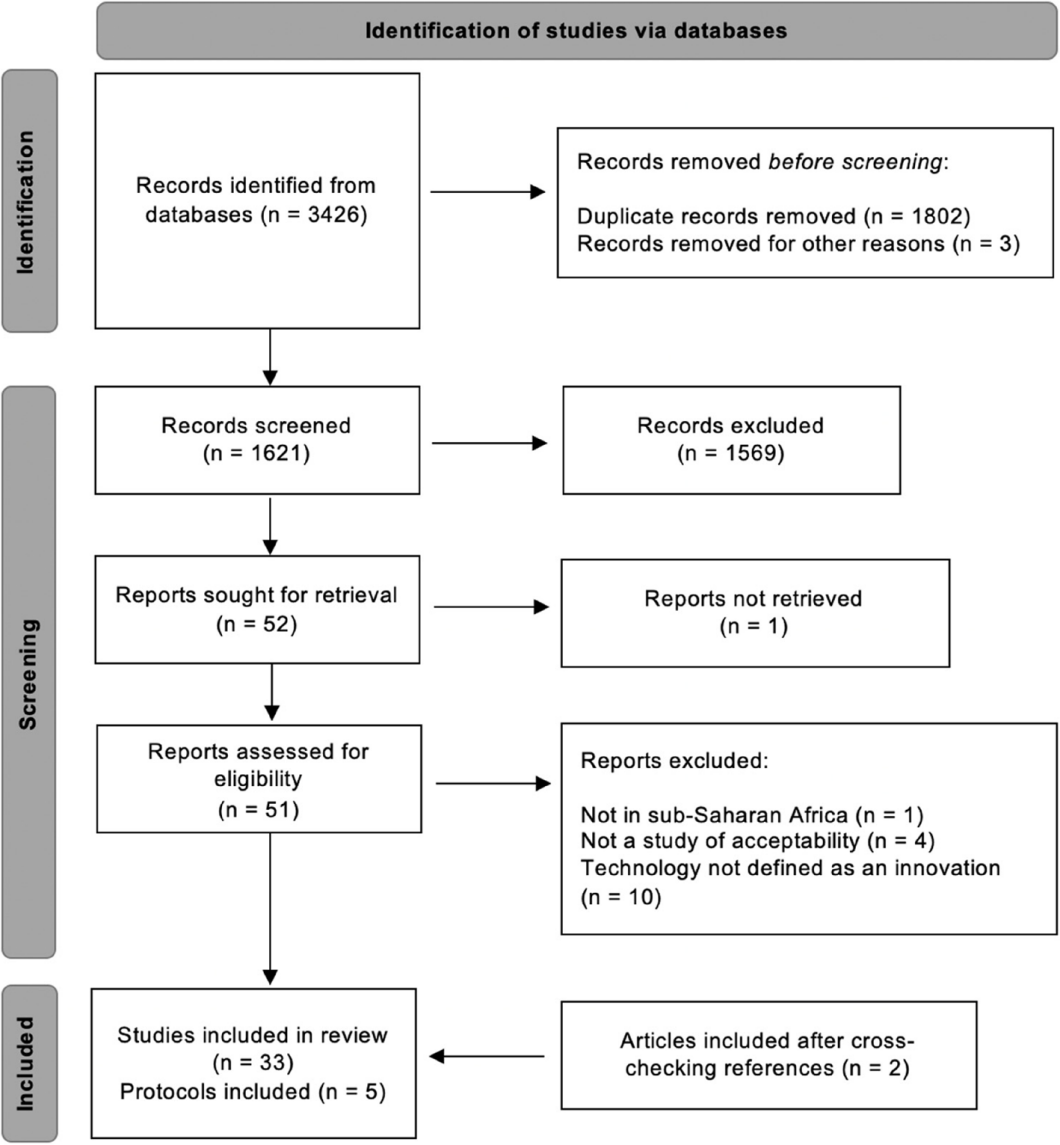
Flow diagram of study selection procedure and results (adapted from PRISMA 2020)

We obtained 3426 results from our database search. We finally included 38 articles for analysis (Fig. 1) [32, 33, 35–70]. Five of the articles were research protocols, while the other 33 presented empirical data. The oldest study was published in 1997, but the majority of studies (over 80%) were published after 2014.

**Fig. 2:**
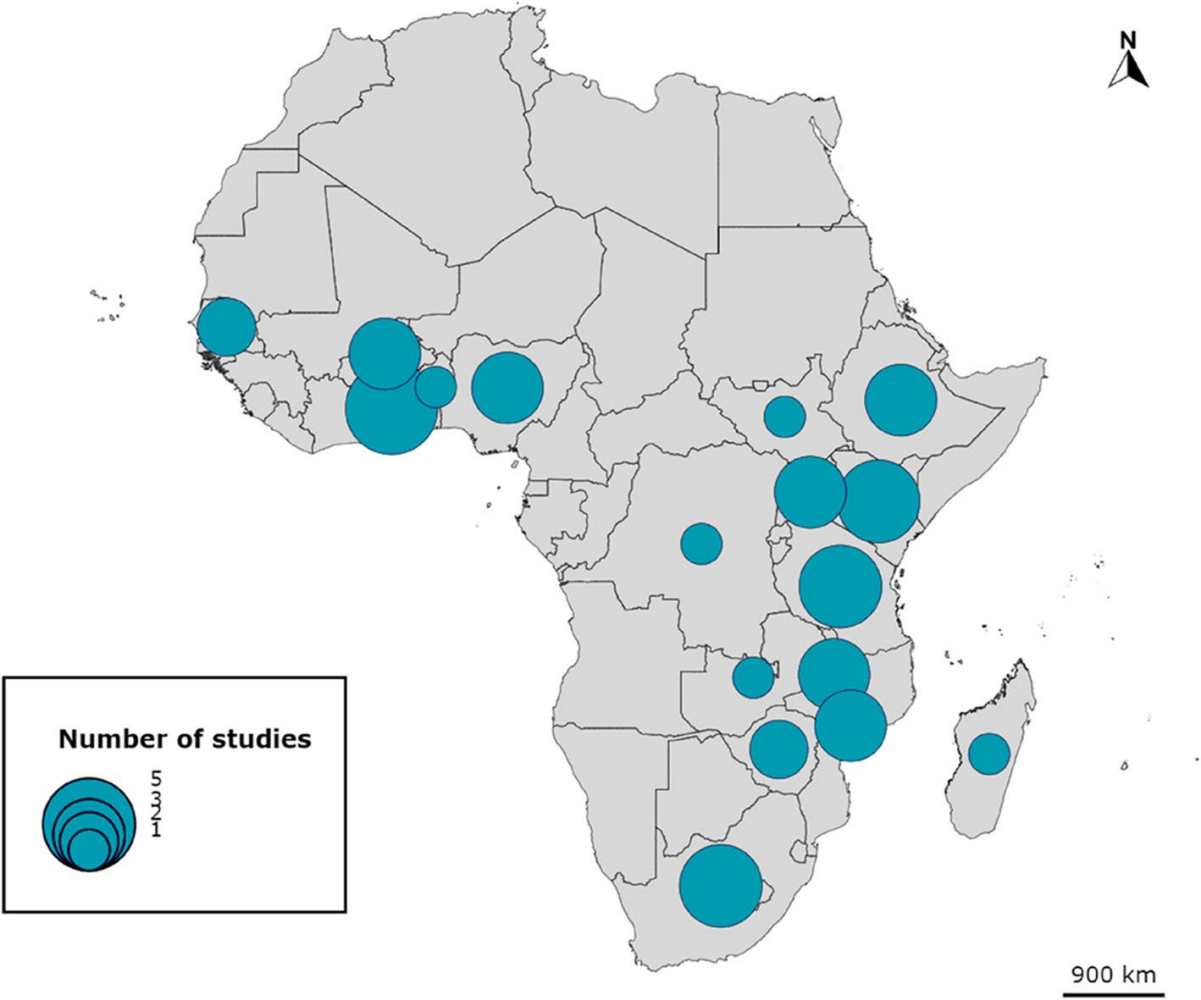
Geographic distribution of studies included in the scoping review

Regarding the geographical distribution of the studies (some studies covered several countries), the country with the most studies is Ghana (*n* = 5), followed by Kenya, South Africa and Tanzania (*n* = 4). A mapping of the studies included is provided in Fig. 2. The setting in which the innovation under study was introduced was rural in 10 articles, urban or peri-urban in 6, both rural and urban in 13, and undocumented in 9.

We have classified the innovations according to their type, the health problem targeted, the target population, etc. (Appendix 3). The three most targeted health problems are maternal and newborn health (*n* = 10), malaria (*n* = 7) and diseases affecting children under five (n = 6).

#### Theories and conceptual frameworks

Of all the articles included (*n* = 38) that aimed to assess the acceptability of a health innovation, only seven defined acceptability (all in different ways) (Table 1).

**Table 1:** Definitions of acceptability used by the authors (*n* = 7/38)

Given definition with the reference of the work on which it is based (if any)	Reference
Study based on the definition of acceptability by Ayala and Elder [71]. They proposed that acceptability refers to determining how well an intervention will be received by the target population and the extent to which the new intervention or its components meets the needs of the target population and organizational setting. The themes included assessing (1) patients' perceived benefits of the innovation, (2) patient satisfaction, and (3) patient preference	[39]
Acceptance comprises positive perceptions, beliefs, and attitudes toward the innovation and the results obtained from the test among users, i.e., health workers and health center attendees	[33]
Acceptability was defined as health workers' positive satisfaction levels and their correct and consistent use of the innovation [72]	[32]
Acceptability by healthcare providers is the factor that affects their willingness to use the innovation during patient interactions. Acceptability by caregivers is the factor that influences their willingness to have the innovation used on their children	[54]
Acceptability and beliefs for Health Surveillance Assistants (HSAs) are their overall perceptions of the innovation and trust in the innovation to correctly direct clinical management. Acceptability and beliefs for caregivers are their views on technology, their level of trust in the innovation, and any concerns about the intervention. (These definitions are linked in the article with some of the CFIR constructs (Consolidated Framework for Implementation Research))	[57]
Acceptability is based on users' perceptions of the different innovations used; caregivers' perceptions of, interaction with, and reaction to the devices when used on children	[58]
Acceptability was determined by evaluating skilled birth attendants' comfort and confidence using the innovation while managing a patient in labor	[67]

Only five papers used a theory or framework to measure and understand acceptability (Table 2). These were used a priori (to guide data collection) in four articles and a posteriori (to guide data analysis) in one. The notion of acceptability was often assimilated or swapped with other concepts such as perception, satisfaction, adoption, use, willingness to use, etc. Many articles present results relative to the acceptability of a health innovation without even defining precisely what they mean by acceptability and therefore what was being studied. The results of our scoping review confirm the lack of clear theoretical or conceptual definitions and foundations for measuring and empirically understanding the acceptability.

**Table 2:** Theories or conceptual frameworks used to measure acceptability

Theory/framework used	Dimensions	Reference
Technology Acceptance Model (TAM) and its extension, TAM2 [23, 73]	Result demonstrability, output quality, job relevance, image, subjective norm, experience, voluntariness, perceived usefulness, perceived ease of use, intention to use, usage behavior	[39, 50]
Concepts of adoption theory [6] and behavior change theory [74]	Complexity, compatibility, relative advantage, observability, trialability	[46]
A conceptual framework that was adopted from previous work on technology acceptance [75, 76] and developed by Asiimwe et al. [33] and Ansbro et al. [32]	Learnability, efficiency, satisfaction, suitability, willingness, effectiveness	[32, 33]

#### Assessment of acceptability

To measure and understand acceptability, 15 studies used qualitative methods, six used quantitative methods, eight used mixed methods, and for nine articles the method was not specified. The authors documented most often the opinion of health workers (*n* = 29; some studies had several target populations), followed by those of patients for 21 of the studies. Finally, the acceptability of managers (*n* = 7), stakeholders in general (*n* = 4), community members who were not necessarily patients at the time of the study (*n* = 3), other medical staff (pharmacists, laboratory technicians, etc.) (*n* = 3), and researchers (*n* = 1) was also considered. In two studies, the innovation was considered acceptable, but it was not clear for whom this acceptability was assessed. In the majority of articles that presented results, acceptability was described as generally positive.

#### Chronology of the acceptability study

**Fig. 3:**
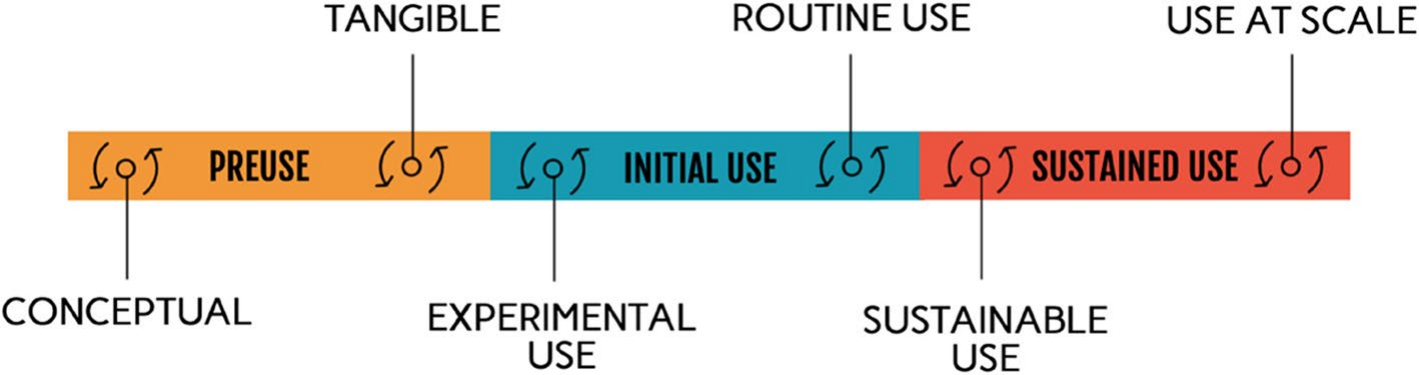
Different stages to measure the acceptability of health technologies

Theoretical articles on acceptability in the literature distinguish between three main stages in acceptability: before the introduction (or before use) of an innovation, during its introduction (or initial use), and after its introduction (or sustained use) [17, 21]. Based on the included studies, we were able to distinguish, for each of these three phases, two potential sub-phases in the acceptability process (Fig. 3). Depending on the period in which acceptability is studied, the influencing factors will not have the same impact on acceptability. Nevertheless, not all of these times are necessarily experienced for each of the innovations introduced (for example, sometimes the innovation is introduced directly into the care routine, or there is no scaling up).

First, at the “conceptual” time, acceptability is measured after explaining the idea or concept of the innovation, but before its introduction or physical presentation. For example, De Haan and al. [46] measured the acceptability of a tool only after giving the participants an information sheet that explained the device. The second stage, the “tangible” (or concrete) time, is a measure of acceptability after having physically presented the innovation but before it was used and integrated into practices. For example, in a study about a new tool for injecting vaccines, the authors stated: “study staff injected vaccine into oranges to demonstrate CPAD [Compact, prefilled, autodisable device] use, as CPAD is not yet licensed for delivery of pentavalent vaccine in the study countries. […]” [44]. The third stage is the “experimental use”. It is when acceptability is measured while the innovation is being used, but not in the way it should normally be used. For example, Ginsburg et al. [55] explain that “we wanted to ensure the application was not used in the clinical care of the patient and that it did not influence the care of the patient in any way. Therefore, we set the oxygen saturation to read 99 percent at all times”. Then, when the innovation is integrated into the care routines, as it should be used normally, it is the time of "routine use". This is the case of a study conducted in Ghana where the acceptability of malaria rapid diagnostic tests (RDTs) was measured after their introduction into care routines [35]. We have identified a fifth time, the time of "sustained use". There, acceptability is measured when the innovation is integrated into care routines during an intervention, but the support and implementation phases are over. For example, Jensen et al. [38] show differences in responses to acceptability questionnaires that were administered before and at the end of the implementation of the intervention. Finally, the last moment of measurement is the time of “scaling up”, when the innovation is diffused beyond its initial place of introduction. The article by Ansbro et al. [32], for example, shows a relative drop in acceptability after the scaling up of a syphilis point-of-care test: “93.8% of pilot HCWs [health care workers] (15/16) and 62.5% of rollout HCWs (15/24) thought patients were somewhat or very accepting of the RST [rapid syphilis test]”. In the context of a scale-up, we can see both the effect of time (evolution between the different phases of the intervention until scale-up) and of the characteristics of the intervention (who implements the intervention and how) on the acceptability of the technology.

### Building a new framework with the best fit framework synthesis

**Fig. 4:**
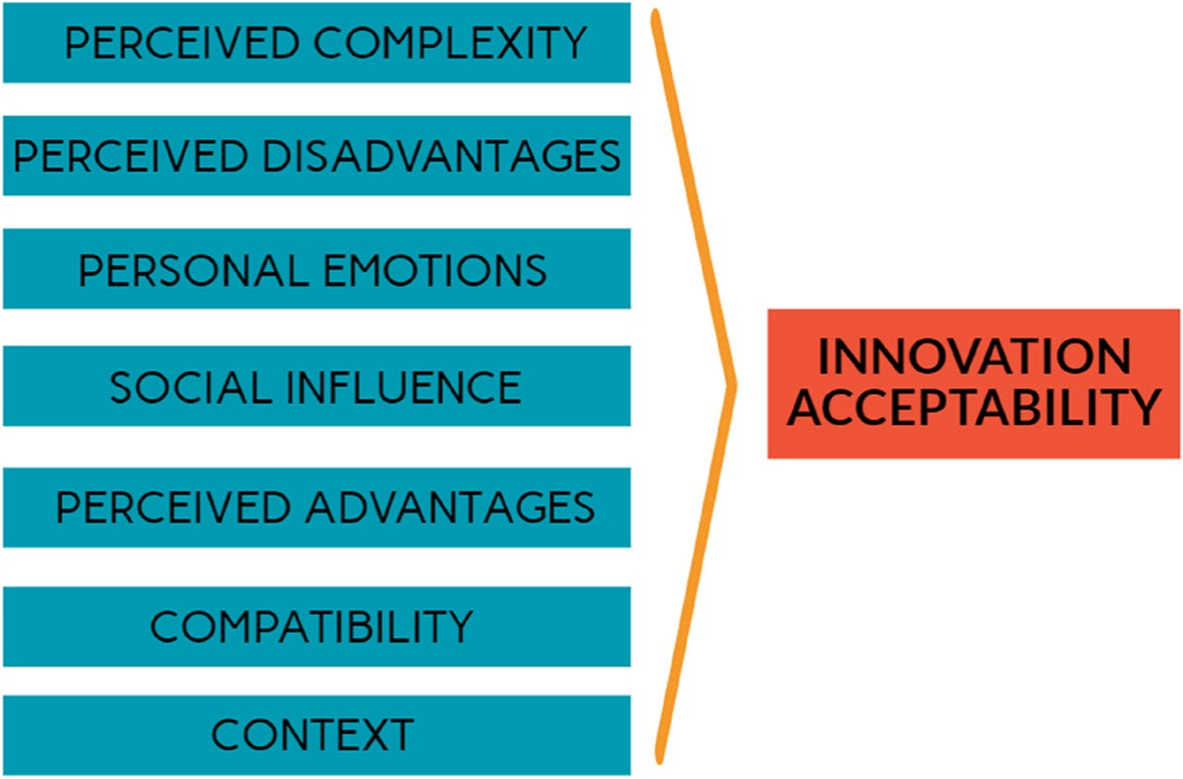
A priori framework

Based on the results of the scoping review, we followed the methodology of the best fit framework synthesis presented earlier [30, 31] in order to build a new framework. We drew on five conceptual frameworks and theories of acceptability [6, 21, 23, 32–34] and began by building an "a priori framework" (Fig. 4). It comprises seven dimensions: perceived complexity, social influence, compatibility, perceived advantages, perceived disadvantages, personal emotions and context. The last theme, 'context’, was supported by only one of the frameworks [32]. This shows the lack of attention to and conceptualization of contextual elements [17], although they are often described as fundamental to understanding acceptability [14].

We then compared the a priori framework to the empirical data of the articles included in our scoping review [30, 31], excluding protocols without empirical results (*n* = 5) so the remainder of the article is based on the analysis of 33 articles. This comparison validated the dimensions of the a priori framework. We found the perceived complexity dimension in 22 out of 33 articles, perceived disadvantages in 23, personal emotions in 21, social influence in 7, perceived advantages in 28 and compatibility in 8. The empirical data also allowed us to redefine the influence of the context dimension and add a dimension related to the intervention.

While in the a priori framework, the context was considered as one of the seven dimensions capable of influencing acceptability directly, the evidence from the empirical studies suggested that context should be understood as a set of elements that can influence each of the other six determinants of acceptability. We also added another specific dimension of influence linked to the intervention itself. Indeed, how the intervention introducing an innovation was designed, implemented, evaluated, and by whom, may influence the acceptability of that technology. For example, an intervention based on randomization between participants may not be accepted (it may result in a refusal to participate), not necessarily because the treatment itself is not accepted but because the form of evaluation is not [19, 77]. Similarly, whether the innovation is introduced by a non-governmental organization or through state support, for example, influences the form of the intervention and may influence the level of acceptability. We found evidence to support these arguments in the studies, as we will see in the next section.

**Fig. 5:**
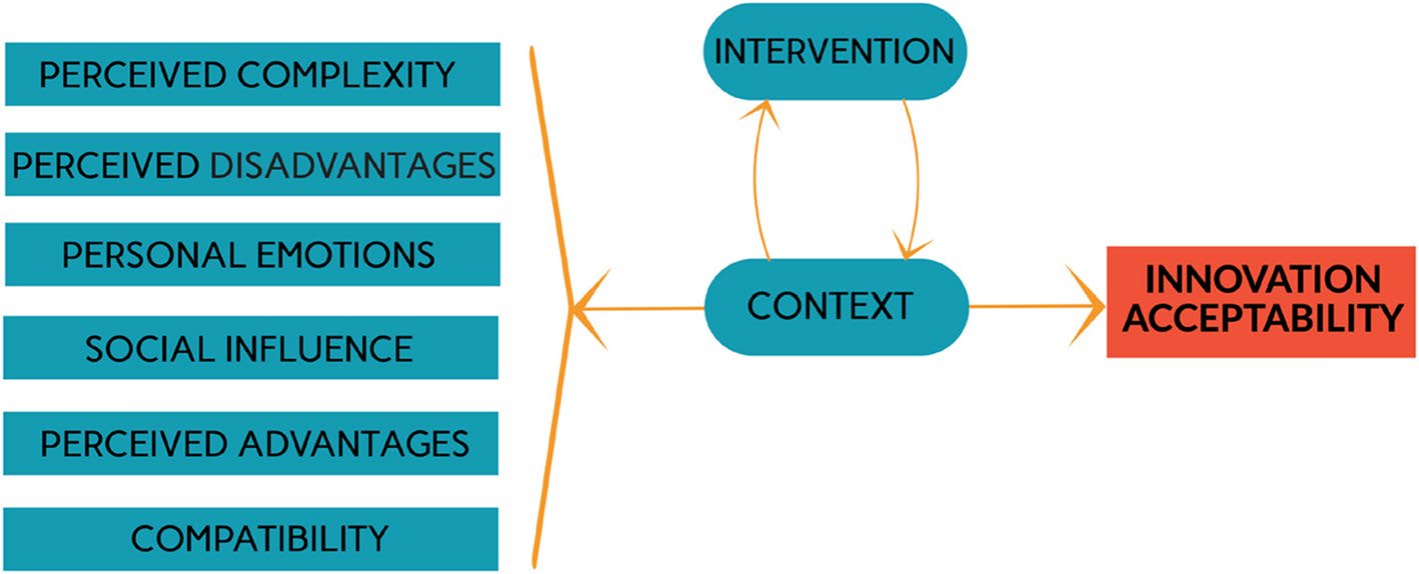
New framework for acceptability of health technological innovation

The empirical data also helped us to identify the type of relationship between the different dimensions and acceptability. Indeed, our conceptual framework is built to highlight causal links between influencing variables and the level of acceptability. We constructed the relationships between acceptability and the six dimensions as "a moderated causal relationship" [78]. The strength and outcome of this relationship (positive, neutral or negative influence) will be moderated by the context and the form of the intervention, considered here as "moderators" or "mediating variables" [78]. The specific link between context and interventions has been little studied in the literature. Nevertheless, on the basis of a few articles [79, 80], we concluded that this link is more of a bidirectional causal relationship [78] since the two variables can influence each other. Indeed, the activities of the intervention will take place in a particular context to which they will have to adapt constantly, and the context itself will evolve following the implementation of the program activities [81, 82]. Thus, starting with the five existing frameworks, then modifying the link between context and other elements and adding the impact of the form of the intervention on the acceptability of the technology, we ended up with the new framework presented in Fig. 5. We will now give more details on each of the dimensions of the framework.

#### Perceived complexity

We defined perceived complexity as the degree to which an innovation and the behavior(s) required to use it are perceived as difficult to understand and to use/perform. In several articles, we found that health workers thought that: "the technology was very easy or easy to use, all mentioning the limited training required to be able to use the device" [49]. The perceived ease of use has a positive impact on the acceptability of the technology. On the contrary, in other studies, some health workers perceived difficulties in using the innovation: "they did not always act on the lights because they were confused. They felt that the yellow light was reported too frequently when patients appeared well” [45]. Here, experience plays an important role in relation to perceived complexity, highlighting the importance of documenting the timing of the acceptability study, as noted earlier: "HCPs [health care providers] struggled to identify how to change their answer selections but were later able to self-correct with practice" [55].

It is also important to compare the perceived understanding with the actual understanding of the technology. For example, the malaria RDT was considered by the patients as "a generic test able to identify any cause of illness, not just malaria, and expectations that a test should result in a diagnosis, even following a negative result" [35]. Another article points out that some patients wanted to receive antimalarials despite a negative RDT result [33]. Here, healthcare professionals may be pressured by patients to obtain a diagnosis or treatment that technology alone cannot provide. Thus, a poor understanding of the technology, even if not necessarily perceived (respondents thought they understood the innovation), can negatively influence the acceptability of the innovation.

#### Perceived disadvantages

Perceived disadvantages are the amount of effort, risk and cost perceived to be associated with an innovation and its use. These disadvantages can be very diverse, such as increased clinical costs per febrile illness episode [46], painfulness [48], concerns about the safety and hygiene of the device [50], increased administrative constraints [53], increased workload [55], etc. All these disadvantages are distinct from the complications linked to the complexity of using the innovation (perceived complexity). They are the potentially harmful effects caused by the use of the innovation and its integration into routines, and influence the acceptability of the technology negatively. On the contrary, the perception that the use of the innovation will cause very few difficulties and complications for users and beneficiaries can influence acceptability positively.

#### Personal emotions

We define this dimension as the emotions an individual feels about an innovation and its use. Some articles have reported the "enthusiasm" of health workers using the new tool [33] or the "enjoyment" of patients participating in a consultation using the technology [47]. Regarding the introduction of a new diagnostic test, while 90% of participants reported that "they valued or appreciated the rapid test", among those who refused the technology, "the more common reasons for refusing the skin snip biopsy were that they 'did not like the idea'." [48]. This clearly shows the influence of personal feelings on acceptability. The technology can provide a "sense of relief" [49], can be judged "likeable" [50], or a "satisfying experience" [53]. Patients may be enthusiastic about the use of new technologies in health care: "when you use it on the child, the mother becomes excited. She sees that we are doing something new on the child and for her it means the facility is improving" [54]. On the contrary, some may feel fear, related to the pain that the tool could cause [33], or to the fear that the tool will be used for purposes other than those announced [69]. It may also upset the patients' certainties: "the RDT results challenged the patient's medical knowledge and brought into question their (presumed) control over their family's health" [62]. Depending on how people feel about the technology, this can have a positive or negative impact on acceptability.

#### Social influence

We defined social influence as the extent to which other people's opinions influence one's degree of acceptability. This dimension took several forms in the empirical data of the articles. From the patients' point of view, the influence exerted by health workers, by their status, was noted: "many respondents conveyed their role as a passive recipient of care, entrusting the clinician with the responsibility to know and prescribe what was 'right' for them. The enactment of this role, coupled with expectations of clinical authority, meant clinical decisions should not be questioned by the patient" [35]. The influence of health authority support was also sometimes highlighted as a positive influence, in contrast to technology manufacturers’ discourse: "Governmental endorsement […] was deemed crucial to acceptability. Congolese practitioners would not perceive evidence provided by other parties, such as manufacturers, as reliable" [46]. Peer influence also plays a role: "Some health workers are not regularly using the CDSS [clinical decision support system] during patient care, which demoralizes other providers who are using the system" [60]. Thus, the opinion of certain key individuals can have both a positive and negative influence on the acceptability of the technology. This influence depends on the opinion of these individuals or institutions on the technology, but also on the importance given to this opinion by the people whose acceptability is being measured.

#### Perceived advantages

Perceived advantages are the extent to which an innovation is perceived as bringing benefits. This is one of the dimensions most commonly found in the results of the studies included in the scoping review. Some of the perceived advantages include the improved diagnostic capacity and prescription of the right treatment [35], improved patient confidence in health workers [33], reduced consultation time [42], simplification of medical procedures [54], the reduction of unnecessary drug prescriptions [69], etc. All these benefits associated with the introduction of the new technology positively influence its acceptability and encourage its use and integration.

However, sometimes the new technology may seem to offer comparatively fewer benefits than what was previously used, which may have a negative impact on its acceptability. For example, some health workers pointed out that the RDTs for malaria (not able to quantify malaria parasites) were less accurate than the laboratory tests used in the past [33].

#### Compatibility

Compatibility is the extent to which an innovation is perceived as being consistent with the existing values, past experiences and needs of potential adopters. This dimension is, therefore, necessarily linked directly to the context. In some studies, the influence of the adequacy between the technology introduced and the setting of its introduction has indeed been highlighted: "the device is considered to have limited compatibility with equipment and competences of Congolese practitioners, especially of those active in rural settings" [46]. Here, the lack of compatibility between the technology introduced and the skills and case management possibilities influence the acceptability of the innovation negatively. Other elements may have an effect, such as its compatibility with the existing work organization in the health center. As pointed out in the article by Jensen et al. [38], in their case, the technology integrates well with the existing system: "eIMCI (electronic Integrated Management of Childhood Illness) was easy to accommodate within the daily staff allocation and clinic workflow". Compatibility can also be related to the perception of the tool itself: "Some of these interviewees said that needle length standards are defined for children of high-income countries" [37], or the adequacy with local values and customs, as underlined by this example of a technology using a blood test: " 'I need my husband to allow me to give the child's blood for testing.' […] 'my religion does not permit us to give blood.'" [33]. However, while this compatibility is a dimension that seems to be of important consideration, we found little referring to it in the articles.

#### Context

We described the ‘context’ dimension as how the contextual environment (organizational, political, economic, social, etc.) influences the degree of acceptability of an innovation. While there was little mention of this dimension in the frameworks used to shape the a priori framework, we found many elements about it in the articles. As explained, we have included the context as a dimension that can impact the influence that each of the factors described above has on acceptability. For example, in a context where the health centers are highly attended, health workers "often noted that once the facility reached around 4 clients per SBA [skilled birth attendants], timely data entry was difficult", thus fueling the perceived disadvantages associated with the system [43]. Similarly, shortages of medicines and medical equipment [60], poor access to electricity in health centers [54], unclear national guidelines [33], or other elements related to the context can also feed the dimension on perceived disadvantages (respectively: impossibility of following the prescription recommendations provided by the innovation; difficulty in charging the device battery; confusion about procedures to follow, etc.).

Another element of the context that can influence acceptability, in particular via the "perceived complexity" dimension, is the level of familiarity with this type of technology or technologies in general. One article notes that the fact that point-of-care tests are regularly performed in health centers was positive because "All health workers agreed that the rapid test was easy to use due to the similarity with the HIV testing" [53]. Similarly, health workers who are familiar with mobile technologies may find a mobile application easier to use than others: "HCPs who did own an android phone demonstrated the ability to move through the application relatively quickly" [55]. On the contrary, unfamiliarity with technologies can have a negative influence, at least at the beginning: "Most HCPs were unfamiliar with touch-screen technology and thus were initially hesitant to use the device" [55].

Regarding perceived advantages, one study explains that in a setting where there is a "shortage of ophthalmic workforce", the new technology could help to overcome this lack of available staff by enabling task shifting and better human resource management [39].

Context can thus influence acceptability through all dimensions. These different examples clearly show the importance of studying the context in which the innovation is introduced and documenting the effects of the context on each of the determinants of acceptability.

#### Intervention

Elements from the articles also led us to add the ‘intervention’ dimension (how an innovation is introduced) in our framework. Indeed, it is important to document and take into account how the intervention was designed, implemented, evaluated and by whom, as this can influence the acceptability of the innovation introduced. As previously discussed, this influence is also directly related to the context [81, 82].

For example, the type of person designated to use the innovation and the place where it is recommended to be used can influence its acceptability: "Using MAP [microarray patch] during outreach activities at fixed posts was also well anticipated by the majority of participants (average of 87.7%) […]. Using MAP at home during outreach strategies was perceived more cautiously (average of 60.2%)" [44]. Other elements related to the form of intervention, such as the role played by the monitoring team and the emphasis placed on the quality assurance program throughout the study [48], whether the intervention was included in a grant program [46], how the technology training was carried out and by whom [32], the quality of the instructions given both to health workers [54] and patients [54] before using the technology, the level of supervision of the intervention [53], etc., can all influence acceptability.

Therefore, how the intervention was shaped and implemented can influence acceptability both positively and negatively. It is suggested, however, that the high level of involvement of both health workers and the local population in the design and management of the program can enhance its acceptability: "They suggested that training the local population to run the program will overcome any potential obstacles related to acceptability and sustainability. Patients saw the importance of community participation as key to building trust and confidence in the program and put the population at ease" [39].

## Discussion

To our knowledge, this study is the first to propose a conceptual framework for studying the acceptability of health innovations in sub-Saharan Africa. To date, the existing literature has emphasized the lack of definition and conceptualization of acceptability [16, 83]. We chose to understand acceptability as a concept made up of multiple dimensions, which help to understand or predict the implementation (if acceptable) or, on the contrary, the de-implementation (if not acceptable) [84] of a health innovation. We believe that our research could help guide researchers, practitioners or anyone who wish to understand the acceptability of an innovation. Our study provides both a timeline of the different times of acceptability, but also a conceptual framework on which different data collection tools, both quantitative and qualitative, can be based in order to document the complex processes of acceptability. Our aim was to provide practical ways of evaluating and understanding acceptability and not merely to think about how to conceptualize this mechanism.

One element that we have not yet addressed, but which seems important to discuss, is the scope of relevance of the framework for the different types of respondents: is there a difference between the factors determining the acceptability of patients and health professionals? In the literature on acceptability, there is often a focus on patients’ acceptability and not on health workers’ acceptability [14], as acceptability is often understood as one of the dimensions of access to care from the patient’s perspective [85, 86]. However, in our study, most technological innovations are used by health workers, so we noticed a reverse trend. It was more often the users of the innovation, i.e. the health workers, who were interviewed. The impact of the different dimensions on acceptability could vary depending on whether the respondent is a health worker using the tool or a patient on or for whom the tool is used. For example, it is more common to measure acceptability in patients through emotional components (in this paper *personal emotions*), whereas, for health professionals, acceptability is more often understood through more cognitive and technical dimensions (*perceived advantages* and *perceived complexity*) [87]. However, the frameworks mobilized and the empirical data from the articles allowed us to hypothesize that, although the strength of their impact on the degree of acceptability may vary, the dimensions of influence that constitute the framework appear to be similar. We believe that the framework can and should therefore be used for all categories of respondent that are directly related to the use of the tool, but it is necessary to confirm this and to study the differences in the influence of the dimensions on acceptability according to the categories of respondent.

Secondly, it might be worthwhile discussing the effect of innovation type on acceptability. In particular, the degree of novelty of the innovation compared to the existing ones, e.g. whether the innovation is radical (fundamental change to the existing) or incremental (minor improvements) [88] can influence its acceptability. For example, an incremental innovation may be perceived as less complex and less risky than a radical innovation (low *perceived complexity* and *perceived disadvantages*), but also as offering fewer advantages compared to the previous system (low *perceived advantages*). Moreover, in the health field, innovations are often linked to scientific advances in clinical or fundamental research. They are, therefore, introduced with the idea that they will necessarily bring added value to the health of populations and their use is therefore often "imposed" on health workers and patients via a top-down approach [89]. All these elements related to the type of innovation studied can influence acceptability and would be useful to study through our framework. Furthermore, in this work, we have focused our research on the empirical data regarding a certain type of innovation (used within the patient-health worker relationship, technological innovations, etc.). It would be advantageous to see if this framework could potentially be extended or adapted to other types of innovation (used by patients or doctors for themselves, social innovations, etc.).

When constructing our framework, since it was initially developed with the aim of collecting empirical data in the context of a West African project, it seemed important to draw on a wide range of theoretical and empirical elements and not only data from high-income countries. It was important to try to avoid ethnocentric bias in implementation studies [90] and a "one-way transfer" of theories, frameworks and experiences from the North to the South [91, 92]. However, none of the frameworks found in the existing literature were initially built specifically on data from LMIC. Consequently, we sought to mobilize empirical research on this topic in sub-Saharan Africa in order to improve existing frameworks. We therefore consider that our framework has benefited from inputs from very different backgrounds and should now be tested to check its suitability in several contexts.

Finally, our study has some limitations. As our scoping review pointed out, acceptability was not necessarily comprehensively and thoroughly understood in most articles. It was sometimes only analyzed as a secondary objective of the research and was often not based on any definition or framework. Thus, the empirical data used to validate and develop the a priori framework may have been limited. For example, other dimensions influencing acceptability, or other links between the different themes, may have been missing from both the conceptual frameworks used for the a priori framework and the empirical data. However, we believe that basing our a priori framework on several different frameworks may have resulted in a robust a priori framework, which may explain its high consistency with the evidence found in the empirical data. In addition, as with all types of systematic reviews, we had to make choices with regard to the databases and keywords used. This may have limited the breadth of empirical data from which we worked on our conceptual framework. It is necessary, therefore, to continue to generate new theoretical perspectives, using the abductive approach that we have initiated here. One of the next steps in this research may also be to consider the construction of indicators and scores out of the different dimensions of this framework. This would allow the development of a ranking and provide a means of measuring acceptability.

## Conclusion

Research on the acceptability of health innovations has lacked the guidance and framing needed to conduct rigorous work, allowing for comparisons and the accumulation of knowledge. Our work on the concept of acceptability, through an abductive approach of confronting frameworks and theories with empirical data, has allowed us to propose a consolidated conceptual framework to support the measurement and understanding of the acceptability of health innovations in sub-Saharan Africa.

### Supplementary Information

**Additional file 1: Appendix 1.** Search strategies for all databases. **Appendix 2.** Construction of the a priori framework. **Appendix 3.** Type of innovation.

## Data Availability

All data generated or analyzed during this study are included in this published article and its supplementary information files.

## Abbreviations

AIRE: Améliorer l’Identification des Détresses Respiratoires chez l’Enfant
CDSS: Clinical Decision Support System
CFIR: Consolidated Framework for Implementation Research
CPAD: Compact, Prefilled, Autodisable Device
eIMCI: Electronic Integrated Management of Childhood Illness
HCP: Health Care Provider
HCW: Health Care Worker
HAS: Health Surveillance Assistant
LMIC: Low- and Middle Income Countries
MAP: Microarray Patch
RDT: Rapid Diagnostic Test
RST: Rapid Syphilis Test
SBA: Skilled Birth Attendant
TAM: Technology Acceptance Model
