# Supplementary material for: Assessing the acceptability of technological health innovations in sub-Saharan Africa: a scoping review and a best fit framework synthesis
Source: BMC Health Serv Res. 2023 Aug 31;23:930. doi: 10.1186/s12913-023-09897-4 (PMC10469465; doi:10.1186/s12913-023-09897-4)
Supplement: Supplementary file 1 — Additional file 1: Appendix 1. Search strategies for all databases. Appendix 2. Construction of the a priori framework. Appendix 3. Type of innovation. [file 12913_2023_9897_MOESM1_ESM.docx]

**Appendix 1 – Search strategies for all databases**

**Pubmed**

((((acceptab*[Title/Abstract]) OR acceptance[Title/Abstract]) OR "Diffusion of Innovation"[Mesh]) AND ((((innovat*[Title/Abstract]) OR technolog*[Title/Abstract]) OR tool*[Title/Abstract]) OR "Biomedical Technology"[Mesh]) AND (((medic*[Title/Abstract]) OR care*[Title/Abstract]) OR health*[Title/Abstract]) AND (((((((((((((((((((((((((((((((((((((((((((((((((((((((((((((((((((((((((((((((((((((((((((((((((("Angola"[Mesh]) OR "Benin"[Mesh]) OR "Botswana"[Mesh]) OR "Burkina Faso"[Mesh]) OR "Burundi"[Mesh]) OR "Cabo Verde"[Mesh]) OR "Cameroon"[Mesh]) OR "Central African Republic"[Mesh]) OR "Chad"[Mesh]) OR "Comoros"[Mesh]) OR "Democratic Republic of the Congo"[Mesh]) OR "Congo"[Mesh]) OR "Cote d'Ivoire"[Mesh]) OR "Djibouti"[Mesh]) OR "Equatorial Guinea"[Mesh]) OR "Eritrea"[Mesh]) OR "Swaziland"[Mesh]) OR "Ethiopia"[Mesh]) OR "Gabon"[Mesh]) OR "Gambia"[Mesh]) OR "Ghana"[Mesh]) OR "Guinea"[Mesh]) OR "Guinea-Bissau"[Mesh]) OR "Kenya"[Mesh]) OR "Lesotho"[Mesh]) OR "Liberia"[Mesh]) OR "Madagascar"[Mesh]) OR "Malawi"[Mesh]) OR "Mali"[Mesh]) OR "Mauritania"[Mesh]) OR "Mauritius"[Mesh]) OR "Mozambique"[Mesh]) OR "Namibia"[Mesh]) OR "Niger"[Mesh]) OR "Nigeria"[Mesh]) OR "Rwanda"[Mesh]) OR ("Sao Tome and Principe"[Mesh])) OR "Senegal"[Mesh]) OR "Seychelles"[Mesh]) OR "Sierra Leone"[Mesh]) OR "Somalia"[Mesh]) OR "South Africa"[Mesh]) OR "South Sudan"[Mesh]) OR "Sudan"[Mesh]) OR "Tanzania"[Mesh]) OR "Togo"[Mesh]) OR "Uganda"[Mesh]) OR "Zambia"[Mesh]) OR "Zimbabwe"[Mesh]) OR "Africa South of the Sahara"[Mesh]) OR Angola[Title/Abstract]) OR Benin[Title/Abstract]) OR Botswana[Title/Abstract]) OR "Burkina Faso"[Title/Abstract]) OR Burundi[Title/Abstract]) OR "Cabo Verde"[Title/Abstract]) OR Cameroon[Title/Abstract]) OR "Central African Republic"[Title/Abstract]) OR Chad[Title/Abstract]) OR Comoros[Title/Abstract]) OR "Democratic Republic of the Congo"[Title/Abstract]) OR "Cote d'Ivoire"[Title/Abstract]) OR "Equatorial Guinea"[Title/Abstract]) OR Eritrea[Title/Abstract]) OR Eswatini[Title/Abstract]) OR Swaziland[Title/Abstract]) OR Ethiopia[Title/Abstract]) OR Gabon[Title/Abstract]) OR Gambia[Title/Abstract]) OR Ghana[Title/Abstract]) OR Guinea[Title/Abstract]) OR Guinea-Bissau[Title/Abstract]) OR Kenya[Title/Abstract]) OR Lesotho[Title/Abstract]) OR Liberia[Title/Abstract]) OR Madagascar[Title/Abstract]) OR Malawi[Title/Abstract]) OR Mali[Title/Abstract]) OR Mauritania[Title/Abstract]) OR Mauritius[Title/Abstract]) OR Mozambique[Title/Abstract]) OR Namibia[Title/Abstract]) OR Niger[Title/Abstract]) OR Nigeria[Title/Abstract]) OR Rwanda[Title/Abstract]) OR "Sao Tome”[Title/Abstract]) OR Senegal[Title/Abstract]) OR Seychelles[Title/Abstract]) OR "Sierra Leone"[Title/Abstract]) OR Somalia[Title/Abstract]) OR "South Africa"[Title/Abstract]) OR Sudan[Title/Abstract]) OR Tanzania[Title/Abstract]) OR Togo[Title/Abstract]) OR Uganda[Title/Abstract]) OR Zambia[Title/Abstract]) OR Zimbabwe[Title/Abstract]) OR “Sub-Saharan Africa”[Title/Abstract]))

**Scopus**

( TITLE-ABS-KEY ( acceptab* OR acceptance) ) AND ( TITLE-ABS-KEY ( medic* OR care* OR health* ) ) AND ( TITLE-ABS-KEY ( innovat* OR technolog* OR tool* ) ) AND ( TITLE-ABS-KEY ( Angola OR Benin OR Botswana OR “Burkina Faso” OR Burundi OR “Cabo Verde” OR Cameroon OR “Central African Republic” OR Chad OR Comoros OR “democratic republic of the congo” OR “Cote d'Ivoire” OR “Equatorial Guinea” OR Eritrea OR Eswatini OR Swaziland OR Ethiopia OR Gabon OR Gambia OR Ghana OR Guinea OR Guinea-Bissau OR Kenya OR Lesotho OR Liberia OR Madagascar OR Malawi OR Mali OR Mauritania OR Mauritius OR Mozambique OR Namibia OR Niger OR Nigeria OR Rwanda OR “Sao Tome” OR Senegal OR Seychelles OR “Sierra Leone” OR Somalia OR “South Africa” OR Sudan OR Tanzania OR Togo OR Uganda OR Zambia OR Zimbabwe OR “sub-saharan africa” ) )

**Web of Science**

TS=(acceptab* OR acceptance) AND TS=(medic* OR care* OR health*) AND TS=(innovat* OR technolog* OR tool*) AND TS=(Angola OR Benin OR Botswana OR “Burkina Faso” OR Burundi OR “Cabo Verde” OR Cameroon OR “Central African Republic” OR Chad OR Comoros OR “democratic republic of the congo” OR “Cote d'Ivoire” OR “Equatorial Guinea” OR Eritrea OR Eswatini OR Swaziland OR Ethiopia OR Gabon OR Gambia OR Ghana OR Guinea OR Guinea-Bissau OR Kenya OR Lesotho OR Liberia OR Madagascar OR Malawi OR Mali OR Mauritania OR Mauritius OR Mozambique OR Namibia OR Niger OR Nigeria OR Rwanda OR “Sao Tome” OR Senegal OR Seychelles OR “Sierra Leone” OR Somalia OR “South Africa” OR Sudan OR Tanzania OR Togo OR Uganda OR Zambia OR Zimbabwe OR “sub-saharan africa”)

**Cochrane library**

TAK : (acceptab* OR acceptance OR "Diffusion of Innovation"[Mesh])

AND TAK : (medic* OR care* OR health* OR "Health"[Mesh])

AND TAK : (innovat* OR technolog* OR tool* OR "Biomedical Technology"[Mesh])

AND TAK : (Angola OR Benin OR Botswana OR “Burkina Faso” OR Burundi OR “Cabo Verde” OR Cameroon OR “Central African Republic” OR Chad OR Comoros OR Congo OR “Cote d'Ivoire” OR “Equatorial Guinea” OR Eritrea OR Eswatini OR Swaziland OR Ethiopia OR Gabon OR Gambia OR Ghana OR Guinea OR Guinea-Bissau OR Kenya OR Lesotho OR Liberia OR Madagascar OR Malawi OR Mali OR Mauritania OR Mauritius OR Mozambique OR Namibia OR Niger OR Nigeria OR Rwanda OR “Sao Tome” OR Senegal OR Seychelles OR “Sierra Leone” OR Somalia OR “South Africa” OR Sudan OR Tanzania OR Togo OR Uganda OR Zambia OR Zimbabwe OR “sub-saharan africa” OR "Africa South of the Sahara"[Mesh])

**Cairn**

Search in abstracts

accepta*

ET innovat* OU technolog* OU outil*

ET santé* OU médic* OU soin*

ET "Afrique du Sud" OU Angola OU Bénin OU Botswana OU "Burkina Faso" OU Burundi OU Cap-Vert OU Cameroun OU Comores OU "Côte d’Ivoire" OU Érythrée OU Éthiopie OU Gabon OU Gambie OU Ghana OU Guinée OU Guinée-Bissau OU "Guinée équatoriale" OU Kenya OU Lesotho OU Libéria OU Madagascar OU Malawi OU Mali OU Maurice OU Mauritanie OU Mozambique OU Namibie OU Niger OU Nigéria OU Ouganda OU "République Centrafricaine" OU Congo OU Rwanda OU "Sao Tomé" OU Sénégal OU Seychelles OU "Sierra Leone" OU Somalie OU Soudan OU Swaziland OU Tanzanie OU Tchad OU Togo OU Zambie OU Zimbabwe

**Scielo**

(acceptab* OR acceptance) AND (medic* OR care* OR health*) AND (innovat* OR technolog* OR tool*) AND (Angola OR Benin OR Botswana OR “Burkina Faso” OR Burundi OR “Cabo Verde” OR Cameroon OR “Central African Republic” OR Chad OR Comoros OR “democratic republic of the congo” OR “Cote d'Ivoire” OR “Equatorial Guinea” OR Eritrea OR Eswatini OR Swaziland OR Ethiopia OR Gabon OR Gambia OR Ghana OR Guinea OR Guinea-Bissau OR Kenya OR Lesotho OR Liberia OR Madagascar OR Malawi OR Mali OR Mauritania OR Mauritius OR Mozambique OR Namibia OR Niger OR Nigeria OR Rwanda OR “Sao Tome” OR Senegal OR Seychelles OR “Sierra Leone” OR Somalia OR “South Africa” OR Sudan OR Tanzania OR Togo OR Uganda OR Zambia OR Zimbabwe OR “sub-saharan africa”)

**Opengrey :**

(acceptab* OR acceptance) AND (medic* OR care* OR health*) AND (innovat* OR technolog* OR tool*) AND (Angola OR Benin OR Botswana OR “Burkina Faso” OR Burundi OR “Cabo Verde” OR Cameroon OR “Central African Republic” OR Chad OR Comoros OR “democratic republic of the congo” OR “Cote d'Ivoire” OR “Equatorial Guinea” OR Eritrea OR Eswatini OR Swaziland OR Ethiopia OR Gabon OR Gambia OR Ghana OR Guinea OR Guinea-Bissau OR Kenya OR Lesotho OR Liberia OR Madagascar OR Malawi OR Mali OR Mauritania OR Mauritius OR Mozambique OR Namibia OR Niger OR Nigeria OR Rwanda OR “Sao Tome” OR Senegal OR Seychelles OR “Sierra Leone” OR Somalia OR “South Africa” OR Sudan OR Tanzania OR Togo OR Uganda OR Zambia OR Zimbabwe OR “sub-saharan africa”)

**EBSCOhost**

TI ( acceptab* OR acceptance ) OR AB ( acceptab* OR acceptance )

AND TI ( medic* OR care* OR health* ) OR AB ( medic* OR care* OR health* )

AND TI ( innovat* OR technolog* OR tool* ) OR AB ( innovat* OR technolog* OR tool* )

AND TI ( Angola OR Benin OR Botswana OR “Burkina Faso” OR Burundi OR “Cabo Verde” OR Cameroon OR “Central African Republic” OR Chad OR Comoros OR “democratic republic of the congo” OR “Cote d'Ivoire” OR “Equatorial Guinea” OR Eritrea OR Eswatini OR Swaziland OR Ethiopia OR Gabon OR Gambia OR Ghana OR Guinea OR Guinea-Bissau OR Kenya OR Lesotho OR Liberia OR Madagascar OR Malawi OR Mali OR Mauritania OR Mauritius OR Mozambique OR Namibia OR Niger OR Nigeria OR Rwanda OR “Sao Tome” OR Senegal OR Seychelles OR “Sierra Leone” OR Somalia OR “South Africa” OR Sudan OR Tanzania OR Togo OR Uganda OR Zambia OR Zimbabwe OR “sub-saharan africa” ) OR AB ( Angola OR Benin OR Botswana OR “Burkina Faso” OR Burundi OR “Cabo Verde” OR Cameroon OR “Central African Republic” OR Chad OR Comoros OR “democratic republic of the congo” OR “Cote d'Ivoire” OR “Equatorial Guinea” OR Eritrea OR Eswatini OR Swaziland OR Ethiopia OR Gabon OR Gambia OR Ghana OR Guinea OR Guinea-Bissau OR Kenya OR Lesotho OR Liberia OR Madagascar OR Malawi OR Mali OR Mauritania OR Mauritius OR Mozambique OR Namibia OR Niger OR Nigeria OR Rwanda OR “Sao Tome” OR Senegal OR Seychelles OR “Sierra Leone” OR Somalia OR “South Africa” OR Sudan OR Tanzania OR Togo OR Uganda OR Zambia OR Zimbabwe OR “sub-saharan africa” )

The equation for EBSCOhost was adapted according to the thesauri available for each database. See below the examples of Academic Search Premier.

**Academic Search Premier**

TI ( acceptab* OR acceptance) OR AB ( acceptab* OR acceptance) OR DE "INNOVATION adoption")

AND TI ( medic* OR care* OR health* ) OR AB ( medic* OR care* OR health* ) OR DE "Health" OR DE "MEDICAL care" )

AND TI ( innovat* OR technolog* OR tool* ) OR AB ( innovat* OR technolog* OR tool* ) OR DE "TECHNOLOGICAL innovations" OR DE "TOOLS" )

AND TI ( Angola OR Benin OR Botswana OR “Burkina Faso” OR Burundi OR “Cabo Verde” OR Cameroon OR “Central African Republic” OR Chad OR Comoros OR “democratic republic of the congo” OR “Cote d'Ivoire” OR “Equatorial Guinea” OR Eritrea OR Eswatini OR Swaziland OR Ethiopia OR Gabon OR Gambia OR Ghana OR Guinea OR Guinea-Bissau OR Kenya OR Lesotho OR Liberia OR Madagascar OR Malawi OR Mali OR Mauritania OR Mauritius OR Mozambique OR Namibia OR Niger OR Nigeria OR Rwanda OR “Sao Tome” OR Senegal OR Seychelles OR “Sierra Leone” OR Somalia OR “South Africa” OR Sudan OR Tanzania OR Togo OR Uganda OR Zambia OR Zimbabwe OR “sub-saharan africa” ) OR AB ( Angola OR Benin OR Botswana OR “Burkina Faso” OR Burundi OR “Cabo Verde” OR Cameroon OR “Central African Republic” OR Chad OR Comoros OR “democratic republic of the congo” OR “Cote d'Ivoire” OR “Equatorial Guinea” OR Eritrea OR Eswatini OR Swaziland OR Ethiopia OR Gabon OR Gambia OR Ghana OR Guinea OR Guinea-Bissau OR Kenya OR Lesotho OR Liberia OR Madagascar OR Malawi OR Mali OR Mauritania OR Mauritius OR Mozambique OR Namibia OR Niger OR Nigeria OR Rwanda OR “Sao Tome” OR Senegal OR Seychelles OR “Sierra Leone” OR Somalia OR “South Africa” OR Sudan OR Tanzania OR Togo OR Uganda OR Zambia OR Zimbabwe OR “sub-saharan africa” )

**Appendix 2 - Construction of the a priori framework**

| **Rogers [1]**  Diffusion of innovation theory | **Sekhon et al. [2]**  Theoretical framework of acceptability of healthcare interventions | **Venkatesh and Davis [3]**  Theoretical Extension of the Technology Acceptance Model | **Huijts et al. [4]**  Technology acceptance framework of new energy technologies | **Adaptation of Jeng [5, 6] by** **Asiimwe et al. [7] and Ansbro et al. [8]**  A conceptual framework for exploring acceptance and use of malaria rapid diagnostic tests Asiimwe et al. [7] and Syphilis Point-of-Care Tests Ansbro et al. [8] |  | **Themes for the a priori framework** |
| --- | --- | --- | --- | --- | --- | --- |
| **Complexity**  is the degree to which an innovation is perceived as difficult to understand and use | **Self-efficacy**  the participant's confidence that they can perform the behaviour(s) required to participate in the intervention  **Intervention coherence**  the extent to which the participant understands the intervention and how it works | **Perceived ease of use**  the extent to which a person believes that using the system will be free of effort | **Perceived behavioral control**  perceived behavioral control refers to the perceived ease or difficulty of performing the behavior  **Perceived costs**  [...] Non-monetary costs are for example effort needed to understand or use the technology | **Learnability**  ability of the adopter to understand how to correctly use the innovation  **Efficacy/efficiency**  ability of the adopters to perform the behavior(s) required to correctly use the innovation and integrate it into their daily routine work  **Satisfaction**  an adopter's feeling that [...] it is a process he/she likes doing |  | **1. Perceived complexity**  The degree to which an innovation and the behavior(s) required to use it are perceived as difficult to understand and to use/perform |
|  |  | **Subjective norm**  a person's perception that most people who are important to him think he should or should not perform the behavior in question  **Image**  the degree to which the use of an innovation is perceived to enhance one's... status in one's social system | **Social norms**  refers to perceived social pressure to perform or not perform the behavior  **Trust**  lack of trust in actors that are responsible for the technology or trust in other parties that are against the technology can decrease acceptance |  |  | **2. Social influence**  The extent to which other people's opinions influence an individual's degree of acceptability |
| **Compatibility**  is the degree to which an innovation is perceived as being consistent with the existing values, past experiences, and needs of potential adopters | **Ethicality**  the extent to which the intervention has a good fit with an individual's value system | **Job relevance**  an individual's perception regarding the degree to which the target system applies to his or her job | **Problem perception**  awareness of problems related to the current system when no new technology is implemented and used | **Suitability**  Adopter's belief that the innovation is relevant for his/her work [...]. |  | **3. Compatibility**  The extent to which an innovation is perceived as being consistent with the existing values, past experiences and needs of potential adopters |
| **Relative advantage**  is the degree to which an innovation is perceived as better than the idea it supersedes  **Observability**  is the degree to which the results of an innovation are visible to others | **Perceived effectiveness**  the extent to which the intervention is perceived as likely to achieve its purpose | **Perceived usefulness**  the extent to which a person believes that using the system will enhance his or her job performance  **Output quality**  how well the system performs the tasks for which it was designed  **Result demonstrability**  the tangibility of the results of using the innovation (observable covariation between usage and positive results) | **Perceived benefits**  can relate to collective benefits but can also comprise personal benefits such as easy access to the technology and improved local conditions | **Suitability**  Adopter's belief that [...] the innovation is likely to truly achieve its purpose |  | **4. Perceived advantages**  The extent to which an innovation is perceived to bring benefits |
| **Triability**  is the degree to which an innovation may be experimented with on a limited basis. [...] An innovation that is trialable represents less uncertainty to the individual who is considering it for adoption, as it is possible to learn by doing | **Burden**  the perceived amount of effort that is required to participate in the intervention  **Opportunity costs**  the extent to which benefits, profits or values must be given up to engage in the intervention |  | **Perceived costs**  Costs of the technology can include personal financial costs such as the costs of purchasing or using the technology or societal costs such as subsidies needed to make the initial investments cost-effective. [...]  **Perceived risks**  risks can include safety risks or uncertain financial costs, such as uncertain repair and maintenance costs |  |  | **5. Perceived disadvantages**  The amount of effort, risk and cost perceived to be associated with an innovation and its use |
|  | **Affective attitude**  how an individual feels about the intervention |  | **Affects (positive or negative)**  affects can concern expected feelings, resulting from the outcome of decisions, or affect when thinking about the technology  **Attitude**  an evaluative integration of cognitions and affects experienced in relation to an object  **Procedural fairness**  procedures are considered to be less fair when people or interest groups have no voice in the decision process  **Distributive fairness**  fairness principles based on collective outcomes (distribution between groups) and personal outcomes  **Personal norm**  personal norms are activated when people are aware of the adverse consequences of not acting in a socially desirable way, and when they feel they can do something to mitigate these problems (as reflected in outcome efficacy) | **Satisfaction**  an adopter's feeling [...] that it is a process he/she likes doing  **Willingness**  the willingness of the adopter to use the innovation and perform the necessary behavior(s) |  | **6. Personal emotions**  The emotions an individual feels about an innovation and its use |
|  |  |  |  | **Effectiveness**  How the organizational and systemic  environment, including implementation of policy, guidelines, supply chain and other logistics, impacted the successful delivery of the program. In addition, how the social context influenced program delivery |  | **7. Context**  How the contextual environment (organizational, political, economic and social) influences the degree of acceptability of an innovation |

**Appendix 3 - Type of innovation**

| Type of innovation | Name | Description | Health issues targeted by the innovation | People targeted by the intervention | Articles |
| --- | --- | --- | --- | --- | --- |
| Diagnostic tool | Malaria rapid diagnostic test (mRDT) | Rapid test that allows parasitological confirmation for malaria | Malaria | All | [7, 9, 10] |
|  |  |  |  | Adult (aged 18 or over), not pregnant, and who just purchased treatment for malaria for him- or herself | [11] |
|  |  |  |  | At three dispensaries - all ages, at the other three dispensaries - only those over five years of age | [12] |
|  | Plasmodium falciparum histidine-rich protein (HRP-II) antigen detection assay (parasight-F test) | Test for malaria diagnosis based on an antigen capture dipstick using a monoclonal antibody to the Plasmodium falciparum histidine-rich protein (HRP-II) | Malaria | All | [13] |
|  | Semi-quantitative pfHRP2 device | Measures of plasma pfHRP2 levels and indicates the likelihood of malaria being the underlying cause of severe febrile illness in parasitaemic African children | Malaria | Children | [14] |
|  | Alere q HIV-1/2 Detect system | Point-of-Care (POC) nucleic acid amplification tests (NAAT) diagnostic for Early Infant HIV Diagnosis (EID) | HIV | HIV-exposed children under 1 year of age | [15] |
|  | Point-of-Care technologies for CD4+ T cell enumeration, hemoglobin and syphilis screening | Integrated package of POC testing services for quantification of hemoglobin and CD4+ T cells, and syphilis serology | HIV, anemia, syphilis | Pregnant women | [16] |
|  | SickleScan | POC screening tool for sickle cell disease | Sickle cell disease | Children, aged 9 months to 5 years, and adults (excluding subjects who had been transfused in the previous 3 months) | [17] |
|  | Ov16 rapid test | Rapid diagnostic tool to detect Igg antibodies to the Ov16 antigen and allow onchocerciasis surveillance | Onchocerciasis | Individuals 5 years and older | [18] |
|  | Lung ultrasound (LUS) | Pneumonia diagnostic technology by analyzing longitudinal and oblique scans of the anterior, lateral and posterior sides of the child's chest | Pneumonia | Children 2 through 23 months of age | [19] |
|  | POC testing for syphilis | Rapid POC test for syphilis screening, integrated into routine antenatal services | Syphilis | Pregnant women | [8, 20] |
|  | Portable Eye Examination Kit (Peek) | Mobile phone-based ophthalmic testing system developed to perform comprehensive eye examinations | Eye Services / ophthalmic services | Adults (the patients ranged in the study from 50 to 77 years) | [21] |
| Improved vaccine's administration | Compact, prefilled, autodisable (CPAD) injection system | CPAD aims to address supply chains and safety issues for vaccines | Vaccination delivery | All but especially children | [22] |
|  | Microarray patch (MAPs) | Array of small solid coated or dissolvable needles that administer a dry formulation of a vaccine or pharmaceutical | Vaccination delivery | Children aged 9-23 months | [23] |
| Voice recognition system | Voice recognition system (VRS) | Uses voice to input data into the computer through a microphone and to reduce the occurrence of prescription errors in pediatric care | Prescription errors | Health workers consulting children | [24] |
| Electronic clinical decision support system | REC (electronic register of consultations), part of the Integrated electronic Diagnosis Approach (IeDA) | REC guides health workers through the IMCI algorithm to improve adherence to the clinical protocol and to provide routine data | Integrated Management of Childhood Illness (IMCI) | Children under five | [25] |
|  | Electronic IMCI (eIMCI) | Improves implementation of IMCI by strengthening health workers' capacity to correctly and comprehensively apply the IMCI guidelines | Integrated Management of Childhood Illness (IMCI) | Children under five | [26] |
|  | Supporting LIFE (SL) electronic CCM application (SL eCCM App) | A mobile health (mhealth) platform for Community Case Management (CCM) | Community Case Management (CCM); childhood diseases | Children under five | [27] |
|  | PANDA (Pregnancy And Newborn Diagnosis Assessment) | Mhealth system created to support community healthcare workers in education, screening, diagnosis, and management decisions with respect to ANC | Maternal health | Pregnant women | [28] |
|  | Electronic clinical decision support system (eCDSS) | Provide step-by-step guidance on what a health worker should observe, ask, and examine during ANC, during labor and after delivery | Antenatal and perinatal care | Pregnant women and newborn | [29, 30] |
|  | Neotree application | A mhealth app that combines immediate digital data capture, evidence-based algorithmic clinical decision support and newborn education on one platform | Newborn health | Newborns | [31] |
|  | mpneumonia | Mobile health application using Android technology that integrates a digital version of the IMCI algorithm with a software-based breath counter and a pulse oximeter | Pneumonia is a primary focus, but it also facilitates the diagnosis and treatment of all childhood illnesses in accordance with IMCI | Young children two months to five years of age | [32, 33] |
|  | E-partogram | Android tablet-based electronic, labor clinical decision support application with algorithms and clinical rules that are based on WHO guidance for managing normal and complicated labor | Maternal and newborn health | Women in labor | [34, 35] |
| Device to monitor one or several medical constants | Wireless fetal heart rate monitoring device | Assess and record fetal heart rate combined with pressure sensors to track uterine contractions to allow real-time assessment of labor | Fetus and mother's health | Pregnant women | [36] |
|  | Doppler fetal heart monitor | Measures the fetal heart rate through an ultrasound probe | Fetus and mother's health | Pregnant women | [37] |
|  | CRADLE vital sign alert (VSA) | A semi-automated device that measures blood pressure, pulse and calculates the mothers' risk of shock | Maternal health | Pregnant women | [38] |
|  | Pulse oximeter with alternative options for power supply | Allows a non-invasive measurement of the oxygen saturation in blood | Childhood illnesses (with a focus on hypoxemia, pneumonia) | Children and babies | [39] |
|  | Pulse oximeters and respiratory rate devices (manual and assisted counters) | Measure oxygen saturation (spo2) and respiratory rate to help the diagnosis of pneumonia symptoms in children | Pneumonia | Children under 5 | [40] |
|  | POC viral load VL assay | POC VL assay (VL quantification is considered the optimal method of monitoring viral suppression) | HIV | Patients initiating ART (adult and pediatric patients) | [41] |
|  | POC urine TFV testing | A rapid POC urine test to assess tenofovir (TFV) levels in urine as a measure for prep (pre-exposure prophylaxis) adherence | HIV | Women (with some medical criteria) | [42] |
|  | E-partograph | Mobile phone used to digitalize paper partograph as electronic (e-Partograph) | Maternal health and newborn health | Pregnant women | [43] |
|  | Eye-tracking | Automated eye-tracking-based tests as a technique for assessing infant cognitive development | Neurocognitive functions | Infants | [44] |

**References**

1. Rogers E. Diffusion of innovation. 1995.

2. Sekhon M, Cartwright M, Francis JJ. Acceptability of healthcare interventions: an overview of reviews and development of a theoretical framework. BMC Health Services Research. 2017;17:88.

3. Venkatesh V, Davis FD. A Theoretical Extension of the Technology Acceptance Model: Four Longitudinal Field Studies. Management Science. 2000;46:186–204.

4. Huijts N, Molin E, Steg L. Psychological factors influencing sustainable energy technology acceptance: A review-based comprehensive framework. Renewable & Sustainable Energy Reviews - RENEW SUSTAIN ENERGY REV. 2012;16.

5. Jeng J. Usability Assessment of Academic Digital Libraries: Effectiveness, Efficiency, Satisfaction, and Learnability. 2005;55:96–121.

6. Jeng J. What Is Usability in the Context of the Digital Library and How Can It Be Measured. Information Technology and Libraries. 2005;24:47–56.

7. Asiimwe C, Kyabayinze DJ, Kyalisiima Z, Nabakooza J, Bajabaite M, Counihan H, et al. Early experiences on the feasibility, acceptability, and use of malaria rapid diagnostic tests at peripheral health centres in Uganda-insights into some barriers and facilitators. Implement Sci. 2012;7:5.

8. Ansbro ÉM, Gill MM, Reynolds J, Shelley KD, Strasser S, Sripipatana T, et al. Introduction of Syphilis Point-of-Care Tests, from Pilot Study to National Programme Implementation in Zambia: A Qualitative Study of Healthcare Workers’ Perspectives on Testing, Training and Quality Assurance. PLoS ONE. 2015;10:e0127728.

9. Ansah EK, Reynolds J, Akanpigbiam S, Whitty CJM, Chandler CIR. “Even if the test result is negative, they should be able to tell us what is wrong with us”: a qualitative study of patient expectations of rapid diagnostic tests for malaria. Malar J. 2013;12:258.

10. Sequeira AR. The Introduction of Rapid Diagnostic Test for Malaria in Mozambique: Local Appropriation and Complementary Therapeutics. Australas Rev Afr Stud. 2015;36:114–28.

11. Liu J, Modrek S, Anyanti J, Nwokolo E, De La Cruz A, Schatzkin E, et al. How do risk preferences relate to malaria care-seeking behavior and the acceptability of a new health technology in Nigeria? BMC Health Serv Res. 2014;14:374.

12. Williams HA, Causer L, Metta E, Malila A, O’Reilly T, Abdulla S, et al. Dispensary level pilot implementation of rapid diagnostic tests: an evaluation of RDT acceptance and usage by providers and patients – Tanzania, 2005. Malar J. 2008;7:239.

13. Mharakurwa S, Manyame B, Shiff CJ. Trial of the ParaSight-F test for malaria diagnosis in the primary health care system, Zimbabwe. Trop Med Int Health. 1997;2:544–50.

14. de Haan F, Onyamboko MA, Fanello CI, Woodrow CJ, Lubell Y, Boon WPC, et al. Exploring health practitioners’ acceptability of a prospective semi-quantitative pfHRP2 device to define severe malaria in the Democratic Republic of Congo. Malar J. 2015;14:503.

15. Dunning L, Kroon M, Hsiao N-Y, Myer L. Field evaluation of HIV point-of-care testing for early infant diagnosis in Cape Town, South Africa. PLoS ONE. 2017;12:e0189226.

16. De Schacht C, Lucas C, Sitoe N, Machekano R, Chongo P, Temmerman M, et al. Implementation of Point-of-Care Diagnostics Leads to Variable Uptake of Syphilis, Anemia and CD4+ T-Cell Count Testing in Rural Maternal and Child Health Clinics. PLoS ONE. 2015;10:e0135744.

17. Nwegbu MM, Isa HA, Nwankwo BB, Okeke CC, Edet-Offong UJ, Akinola NO, et al. Preliminary Evaluation of a Point-of-Care Testing Device (SickleSCAN^TM^) in Screening for Sickle Cell Disease. Hemoglobin. 2017;41:77–82.

18. Dieye Y, Storey HL, Barrett KL, Gerth-Guyette E, Di Giorgio L, Golden A, et al. Feasibility of utilizing the SD BIOLINE Onchocerciasis IgG4 rapid test in onchocerciasis surveillance in Senegal. PLoS Neglected Tropical Diseases. 2017;11.

19. Lenahan JL, Volpicelli G, Lamorte A, Jehan F, Bassat Q, Ginsburg AS. Multicentre pilot study evaluation of lung ultrasound for the management of paediatric pneumonia in low-resource settings: a study protocol. BMJ Open Respir Res. 2018;5:e000340.

20. Bocoum FY, Tarnagda G, Bationo F, Savadogo JR, Nacro S, Kouanda S, et al. Introducing onsite antenatal syphilis screening in Burkina Faso: implementation and evaluation of a feasibility intervention tailored to a local context. BMC Health Services Research. 2017;17.

21. Lodhia V, Karanja S, Lees S, Bastawrous A. Acceptability, Usability, and Views on Deployment of Peek, a Mobile Phone mHealth Intervention for Eye Care in Kenya: Qualitative Study. JMIR Mhealth Uhealth. 2016;4:e30.

22. Guillermet E, Dicko HM, Mai LTP, N’Diaye M, Hane F, Ba SO, et al. Acceptability and Feasibility of Delivering Pentavalent Vaccines in a Compact, Prefilled, Autodisable Device in Vietnam and Senegal. PLoS ONE. 2015;10:e0132292.

23. Guillermet E, Alfa DA, Phuong Mai LT, Subedi M, Demolis R, Giersing B, et al. End-user acceptability study of the nanopatch^TM^; a microarray patch (MAP) for child immunization in low and middle-income countries. Vaccine. 2019;37:4435–43.

24. Migowa AN, Macharia WM, Samia P, Tole J, Keter AK. Effect of a voice recognition system on pediatric outpatient medication errors at a tertiary healthcare facility in Kenya. Ther Adv Drug Saf. 2018;9:499–508.

25. Blanchet K, Lewis JJ, Pozo-Martin F, Satouro A, Somda S, Ilboudo P, et al. A mixed methods protocol to evaluate the effect and cost-effectiveness of an Integrated electronic Diagnosis Approach (IeDA) for the management of childhood illnesses at primary health facilities in Burkina Faso. Implement Sci. 2016;11:111.

26. Jensen C, McKerrow NH, Wills G. Acceptability and uptake of an electronic decision-making tool to support the implementation of IMCI in primary healthcare facilities in KwaZulu-Natal, South Africa. Paediatr Int Child Health. 2019;:1–12.

27. Ide N, Hardy V, Chirambo G, Heavin C, O’Connor Y, O’Donoghue J, et al. People Welcomed This Innovation with Two Hands: A Qualitative Report of an mHealth Intervention for Community Case Management in Malawi. Annals of global health. 2019;85.

28. Benski AC, Stancanelli G, Scaringella S, Herinainasolo JL, Jinoro J, Vassilakos P, et al. Usability and feasibility of a mobile health system to provide comprehensive antenatal care in low-income countries: PANDA mHealth pilot study in Madagascar. J Telemed Telecare. 2017;23:536–43.

29. Duysburgh E, Temmerman M, Yé M, Williams A, Massawe S, Williams J, et al. Quality of antenatal and childbirth care in rural health facilities in Burkina Faso, Ghana and Tanzania: an intervention study. Trop Med Int Health. 2016;21:70–83.

30. Sukums F, Mensah N, Mpembeni R, Massawe S, Duysburgh E, Williams A, et al. Promising adoption of an electronic clinical decision support system for antenatal and intrapartum care in rural primary healthcare facilities in sub-Saharan Africa: The QUALMAT experience. Int J Med Inform. 2015;84:647–57.

31. Crehan C, Kesler E, Nambiar B, Dube Q, Lufesi N, Giaccone M, et al. The NeoTree application: developing an integrated mHealth solution to improve quality of newborn care and survival in a district hospital in Malawi. BMJ Glob Health. 2019;4:e000860.

32. Ginsburg AS, Tawiah Agyemang C, Ambler G, Delarosa J, Brunette W, Levari S, et al. mPneumonia, an Innovation for Diagnosing and Treating Childhood Pneumonia in Low-Resource Settings: A Feasibility, Usability and Acceptability Study in Ghana. PLoS ONE. 2016;11:e0165201.

33. Ginsburg AS, Delarosa J, Brunette W, Levari S, Sundt M, Larson C, et al. mPneumonia: Development of an Innovative mHealth Application for Diagnosing and Treating Childhood Pneumonia and Other Childhood Illnesses in Low-Resource Settings. PLoS ONE. 2015;10:e0139625.

34. Litwin LE, Maly C, Khamis AR, Hiner C, Zoungrana J, Mohamed K, et al. Use of an electronic Partograph: feasibility and acceptability study in Zanzibar, Tanzania. BMC Pregnancy Childbirth. 2018;18:147.

35. Sanghvi H, Mohan D, Litwin L, Bazant E, Gomez P, MacDowell T, et al. Effectiveness of an Electronic Partogram: A Mixed-Method, Quasi-Experimental Study Among Skilled Birth Attendants in Kenya. Glob Health Sci Pract. 2019;7:521–39.

36. Mugyenyi GR, Atukunda EC, Ngonzi J, Boatin A, Wylie BJ, Haberer JE. Functionality and acceptability of a wireless fetal heart rate monitoring device in term pregnant women in rural Southwestern Uganda. BMC Pregnancy Childbirth. 2017;17:178.

37. Bezuidenhout H, Woods D, Wyatt J, Lawn J. Does fetal heart rate count? Developing a low cost, alternative powered Doppler fetal heart monitor for use in low resource high mortality settings. 2006. p. 155–9.

38. Vousden N, Lawley E, Nathan HL, Seed PT, Brown A, Muchengwa T, et al. Evaluation of a novel vital sign device to reduce maternal mortality and morbidity in low-resource settings: a mixed method feasibility study for the CRADLE-3 trial. BMC Pregnancy Childbirth. 2018;18:115.

39. Bezuidenhout H, Woods D, Wyatt J, Lawn J. Are you blue yet? Developing low cost, alternative powered pulse oximetry for ill babies and children. 2006. p. 83–7.

40. Baker K, Akasiima M, Wharton-Smith A, Habte T, Matata L, Nanyumba D, et al. Performance, Acceptability, and Usability of Respiratory Rate Timers and Pulse Oximeters When Used by Frontline Health Workers to Detect Symptoms of Pneumonia in Sub-Saharan Africa and Southeast Asia: Protocol for a Two-Phase, Multisite, Mixed-Methods Trial. JMIR Res Protoc. 2018;7:e10191.

41. Meloni ST, Agbaji O, Chang CA, Agaba P, Imade G, Oguche S, et al. The role of point-of-care viral load monitoring in achieving the target of 90% suppression in HIV-infected patients in Nigeria: study protocol for a randomized controlled trial. BMC Infect Dis. 2019;19:368.

42. Drain P, Ngure K, Mugo N, Spinelli M, Chatterjee P, Bacchetti P, et al. Testing a Real-Time Tenofovir Urine Adherence Assay for Monitoring and Providing Feedback to Preexposure Prophylaxis in Kenya (PUMA): Protocol for a Pilot Randomized Controlled Trial. JMIR Res Protoc. 2020;9.

43. Tadesse Y, Gelagay AA, Tilahun B, Endehabtu BF, Mekonnen ZA, Gashu KD. Willingness to Use Mobile based e-Partograph and Associated Factors among Care Providers in North Gondar Zone, Northwest Ethiopia. Online J Public Health Inform. 2019;11:e10.

44. Forssman L, Ashorn P, Ashorn U, Maleta K, Matchado A, Kortekangas E, et al. Eye-tracking-based assessment of cognitive function in low-resource settings. Arch Dis Child. 2017;102:301–2.
